# Investigating attitudes towards medication and barriers to self-management among Hungarian adults with diabetes mellitus: A cross-sectional study

**DOI:** 10.1371/journal.pone.0317034

**Published:** 2025-03-26

**Authors:** Klára Bíró, Mihály Varga, Viktor Dombrádi, Nóra Kovács, Attila Nagy, Gábor Bányai, Klára Boruzs

**Affiliations:** 1 Institute of Health Economics and Management, Faculty of Economics and Business, University of Debrecen, Debrecen, Hungary; 2 Doctoral School of Health Sciences, University of Debrecen, Debrecen, Hungary; 3 Department of Public Health and Epidemiology, Faculty of Medicine, University of Debrecen, Debrecen, Hungary; 4 Department of Health Informatics, Institute of Health Sciences, Faculty of Health Sciences, University of Debrecen, Debrecen, Hungary; University of Glasgow, UNITED KINGDOM OF GREAT BRITAIN AND NORTHERN IRELAND

## Abstract

The key to effective patient care is the patient’s proper cooperation, so it is important to examine the beliefs about medicine and self-management among diabetes patients. Therefore, the primary aim of the study was to investigate the attitude toward metformin medication and self-management of adult patients with diabetes in Hungary. A total of 591 metformin-taking diabetes patients completed the Beliefs about Medicine Questionnaire, while 283 metformin-taking diabetes patients completed the Environmental Barrier Assessment Scale. Multivariate regression analysis was conducted to investigate which socio-demographic factors influence the beliefs regarding medicines and various environmental barriers to diabetes self-management. Participants who reported a good or very good financial status were more likely to feel the need to take metformin compared to those perceiving bad or very bad financial status (coef = 0.25; p = 0.020). Respondents between 55-64 years and those older than 65 were significantly less concerned about metformin than those aged 18-24 years (coef = -0.47; p = 0.028 and coef = -0.41; p = 0.047). Participants with secondary education were significantly less likely to think that metformin was harmful than those with primary education (coef = -0.50; p = 0.009). In addition, those aged 35 or older saw more barriers to taking medication than those aged 18-24 years (35-44: coef = -0.54; p = 0.020; 45-54: coef = -1.15; p < 0.001; 55-64: coef = -1.06; p < 0.001; 65 years or older: coef = -1.48; p < 0.001). Also, significant negative association was found for several factors (such as age, education, self-reported financial status, subjective health status) with barriers regarding exercise. Overall, socio-demographic factors significantly impact both the attitude toward medicine and diabetes self-management. However, the impact considerably varies according to different beliefs and environmental barriers. To further improve drug adherence and self-management for diabetes, doctors should take into consideration the relevant socio-demographic factors when communicating with their patients.

## Introduction

Diabetes mellitus is one of the most common endocrine chronic diseases, affecting more than 10% of the global population [1,2]. According to forecasts, the number of people with diabetes in the world will reach 643 million by 2030, and 783 million by 2045 [1,3]. The prevalence of diabetes is evenly distributed between the sexes [4]. According to a 2014 research carried out in Hungary, 19% of the population between 60 and 70 years, while 20% of those over 70 years suffer from type 2 diabetes, and it was also highlighted that the incidence of diabetes increases with age [5]. According to this same study, the prevalence of type 2 diabetes is constantly increasing in Hungary. The total number of cases of diabetes in adults was 661,400 in 2021, which represents 9.1% of the total population [5,6].

Metformin is an antidiabetic drug used today as the gold standard for the treatment of type 2 diabetes, insulin resistance, and prediabetes [7]. The increasing prevalence of diabetes worldwide is influenced by a complex interplay of social, economic, demographic, environmental, and genetic factors. The increase in the prevalence of type 2 diabetes and its associated risk factors (obesity, unhealthy diet, and extensive physical inactivity) together cause a further increase in the number of patients [8]. Several factors (patients’ sex, beliefs about medication, duration of illness, and highest education) have been identified to be associated with medication non-adherence in patients with various chronic diseases, including type 2 diabetes [9,10].

While genetics and lifestyle factors play a significant role in the development and treatment of diabetes, the living environment of individuals also plays a decisive role. Environmental barriers, from access to health services to socioeconomic factors, can significantly affect diabetes management [11].

Understanding the environmental barriers and societal attitudes that impact diabetes management is crucial for improving health outcomes and reducing disparities [12].

The relationship between Beliefs About Medicines Questionnaire (BMQ) data and changes in medication adherence has been investigated in a number of studies worldwide [13–18], including high cholesterol [19], diabetes [20,21], hypertension [22–24], rheumatoid arthritis [25–27], epilepsy [28,29], Chron’s disease [30], and asthma [31]. For each study, the questionnaire was suitable for answering the research question for each analysis.

The article’s methodology focused on developing and validating the Environmental Barriers to Adherence Scale (EBAS), a tool designed to measure obstacles diabetic patients encounter in adhering to their care regimens. The insights formed a pool of items, refined to those specifically related to environmental (not social or emotional) barriers, resulting in a 60-item scale with four subscales (diet, exercise, glucose testing, and medication). Additionally, the scale includes 3 exercise-specific barriers and 5 diet-specific barriers [12,28,32]. Each item on the EBAS was rated on a five-point scale (from “never” to “always”), allowing for the calculation of a global barrier score, scores for each subscale, and additional composite scores based on shared items across subscales.[32] Through these steps, the study established EBAS as a valid and reliable tool for measuring environmental barriers in diabetes management, capable of distinguishing specific barriers in self-care areas like medication and diet adherence.

Taking these into consideration, the primary aim of the study was to investigate the attitude toward metformin medication and self-management of adult patients with diabetes mellitus in Hungary with the Beliefs About Medicines Questionnaire (BMQ) and the Environmental Barrier Assessment Scale (EBAS). Another aim was to investigate the internal consistency of all scales in both instruments to determine if the Hungarian translations can be used for research purposes.

## Materials and methods

### Data collection and study settings

A private company (SZLEM Service L.P.) sent the online questionnaires to adult Hungarian citizens. These citizens were part of an online market research panel used to conduct national surveys. The panels were designed to be representative of the population of the country and prior consent was required for membership. The survey started on the 16th January 2023 and ended on the 7th March 2023. Participants were interviewed using an online form that took about 15 minutes to complete. The questionnaire was filled out anonymously. During the survey, the participants signed an informed consent form containing all the details of the study. The patients were informed in written form about the aims of the study and were assured that the participation was voluntary.

The following socio-demographic data were collected: age, sex, education, region (Nomenclature of territorial units for statistics 2 - NUTS 2), town size (number of citizens), marital status, financial situation, being a healthcare worker, and perceived health status. Participants were classified into good or very good, fair and bad or very bad financial situation based on their subjective perception. Participants were also grouped by educational level, such as primary, secondary, and college or university. Overall, 800 adults completed the survey. However, not all of them were taking metformin medication and not all relevant questions of the BMQ and EBAS were answered. Therefore, those not taking the medication or not completing the entire questionnaire were omitted from the study. In the end, 591 answers were included in the analysis regarding BMQ, while 283 regarding EBAS. Before the survey, the study was approved by the Scientific Research and Ethics Committee of the Medical Research Council in Hungary (BMEÜ/2801- 3/2022/EKU). According to the Committee the informed consent complies with the Ministerial Decree in Hungary on medical research involving human subjects.

### Questionnaires

The patients’ beliefs about medicines were measured using the BMQ questionnaire. The validated Hungarian version was used to assess the participants’ positive and negative beliefs, about medicines in general and for metformin specifically [19]. Research in Hungary focused on BMQ-Specific for cholesterol-lowering drugs demonstrated closely aligned values with the current study. Furthermore, studies conducted across the Visegrad Group (V4) countries—Czechia, Slovakia, Poland, and Hungary—showed that Cronbach’s alpha values for Necessity and Concern subscales were consistently high. Previous research assessing the Sinhalese translation of the BMQ reported comparable internal consistency for the overall scale. Similarly, a study on the Portuguese translation of the BMQ found a high Cronbach’s alpha coefficient for the entire questionnaire. For the Polish version among cardiovascular patients, internal consistency was also strong across subscales.[33]

The barriers to self-care, including diet, exercise, glucose testing and medication were assessed using EBAS, which was not translated into Hungarian prior to the study.

The questionnaire did not ask the participants and the research database did not store any personal data, thereby ensuring anonymity. In other words, the personal data handled by the panel on the basis of data management consent for the purpose of contact will not be linked to the answers under any circumstances.

#### Beliefs About Medicines Questionnaire (BMQ).

The BMQ questionnaire was first introduced by Horne et al. [34]. It was initially developed in English in 1999. It contains two short questionnaires: the BMQ-Specific and the BMQ-General questionnaire [34]. The BMQ-Specific was designed to assess key beliefs about influencing interactions with prescribed medications. It consists of two scales: special needs and specific concerns. The special needs (Necessity scale) scale assesses patients’ beliefs about their personal needs for the prescribed medication, as well as their concerns about treatment (Concerns scale). The BMQ-General assesses the social representation of medicines with a two-step assessment of the extent to which a medicine is fundamentally harmful (Harm scale) and the perception of the benefits of over-prescribed addictive medicines (Overuse scale). The BMQ is used worldwide and has been translated and validated into several languages [35,36], and several studies have been conducted on the relationship between BMQ data and changes in medication adherence for chronic diseases [26,33,37–40].

In 2020 the BMQ instrument was translated and validated in Hungarian [19], which was later used to assess cholesterol-lowering medication related attitudes [33].

#### Environmental Barrier Assessment Scale (EBAS).

Several studies [11,12,32] have been conducted to measure self-management among people with diabetes. One such instrument used is the EBAS. This questionnaire consists of 60 items with four scales: diet, exercise, glucose testing, and medication. Each question had five possible answers: never, rarely, sometimes, often, and always. Options were scored from 1 (never) to 5 (always). A higher score indicates a higher barrier [12,32].

The study used concurrent measures, correlating EBAS scores with established scales—the Barriers to Adherence Scale (BAS) and the diabetes-care profile (DCP)—to assess the validity of the EBAS. The internal consistency was measured using Cronbach’s alpha, showing high reliability, and test-retest reliability scores indicated stability over time.[12]

Through these steps, the study established EBAS as a valid and reliable tool for measuring environmental barriers in diabetes management, capable of distinguishing specific barriers in self-care areas like medication and diet adherence.[12]

#### Translation and Language Adaptations.

We used a similar procedure to translate the BMQ-Specific for metformin and EBAS questionnaires into Hungarian. The original English versions [32,34] were translated by two independent professional translators. Once completed, the two translations were merged into a single translation and modified so that the questions focused on metformin-containing medications. After that, as part of the validation process, each translation was tested by ten Hungarian-speaking citizens, and the translations were revised based on their feedback. A third independent translator translated the EBAS questionnaire back into English. The research team deemed these translations to be adequate.

### Data analysis

Categorical variables were expressed as frequency (%), and continuous variables were expressed as mean and standard deviation (SD). Cronbach’s alpha was calculated to assess the reliability of each subscale for both instruments. If the alpha value was greater than 0.70, the internal reliability was considered acceptable [41].

The Necessity and Concerns scores for the BMQ-Specific were divided by the median to create 4 categories: “skeptical”, “accepting”, “indifferent” and “ambivalent”. “Ambivalent” towards metformin-containing drugs agreed that they need metformin-containing drugs. “Accepting” metformin drugs means that they agree with the need. Respondents who are concerned about the need for metformin drugs and the high likelihood of side effects are classified as “skeptical”. Respondents were considered “indifferent” if they were not concerned neither convinced about the necessity of the medication.

Finally, robust regression analysis was performed to evaluate the relationship between socio-demographic variables and the subscales for both BMQ and EBAS. Results are presented as coefficients and corresponding 95% confidence intervals (Cl).

Statistical significance was defined as a p-value of less than 0.05. STATA v13 software (Stata Corp LLC, College Station, TX, USA) was used for data analysis.

## Results

### Demographic characteristics of the participants

A total of 800 patients with diabetes completed the original survey. The average age of total respondents was 51.7 (SD 16.7). Less than half of the participants were women (48.5%), the proportion of men (51.5%) was slightly higher. Most of the participants had a secondary education (58.5%). Most of the respondents lived in the Pest/Budapest region (39.50%) and town size with more than 100,000 inhabitants. The highest proportion of respondents rated their financial situation as fair (57.2%), while the smallest proportion of respondents rated bad or very bad (20.7%) (see Table 1). A total of 591 metformin-taking diabetes patients completed the BMQ questionnaire and 283 completed the EBAS questionnaire. The mean age of BMQ respondents was 52.0 and the average age of EBAS respondents was 45.8. For the BMQ questionnaire, the proportion of women (50.1%) was higher, while for the EBAS questionnaire it was higher for men (56.5%). In both the BMQ and EBAS questionnaires, the majority of participants have a secondary school education. Most of the respondents lived in the Pest/Budapest region (41.6% and 43.5%), and in cities with more than 100,000 inhabitants. For financial situation, similar results were found for both BMQ and EBAS respondents (Fair: 56.5%; Fair: 49.1%).

**Table 1:** Demographic data of the respondents.

		Total		BMQ		EBAS	
		**n = 800**		**n = 591**		**n = 283**	
		**N**	**%**	**N**	**%**	**N**	**%**
**Sex**	Men	412	51.5%	295	49.9%	160	56.5%
	Women	388	48.5%	296	50.1%	123	43.5%
**Age**	Mean, SD	51.7	16.7	52.0	16.3	45.8	14.6
**Education**	Primary school	35	4.4%	20	3.4%	5	1.8%
	Secondary school	468	58.5%	333	56.4%	152	53.7%
	College or university	297	37.1%	238	40.3%	126	44.5%
**Region (NUTS 2)**	Pest/Budapest	316	39.5%	246	41.6%	123	43.5%
	Central Transdanubia	84	10.5%	66	11.2%	31	11.0%
	Western Transdanubia	52	6.5%	42	7.1%	17	6.0%
	Southern Transdanubia	62	7.8%	41	6.9%	17	6.0%
	Northern Hungary	82	10.3%	56	9.5%	25	8.8%
	Northern Great Plain	111	13.9%	68	11.5%	33	11.7%
	Southern Great Plain	93	11.6%	72	12.2%	37	13.1%
**Town size (number of citizens)**	<1,000	32	4.0%	24	4.1%	13	4.6%
	1,000-1,999	50	6.3%	30	5.1%	10	3.5%
	2,000-4,999	103	12.9%	72	12.2%	29	10.3%
	5,000-19,999	149	18.6%	101	17.1%	45	15.9%
	20,000-99,999	180	22.5%	136	23.0%	59	20.9%
	≥100,000	286	35.8%	228	38.6%	127	44.9%
**Marital status**	Married/in relationship	544	68.0%	415	70.2%	212	74.9%
	Other	256	32.0%	176	29.8%	71	25.1%
**Self-reported financial situation**	Good or very good	173	22.1%	141	24.4%	102	36.6%
	Fair	448	57.2%	326	56.5%	137	49.1%
	Bad or very bad	162	20.7%	110	19.1%	40	14.3%
**Healthcare worker**	Yes	97	12.1%	69	11.7%	39	13.8%
	No	703	87.9%	522	88.3%	244	86.2%
**Subjective health status**	Good or better	491	61.4%	358	60.6%	187	66.1%
	Fair or poor	309	38.6%	233	39.4%	96	33.9%

SD: Standard deviation, N: number of respondents, BMQ: Beliefs About Medicines Questionnaire, EBAS: Environmental Barriers to Adherence Scale, NUTS: Nomenclature of territorial units for statistics

Regarding the BMQ-Specific questionnaire Necessity scale, most respondents agreed (60.7%) with the statement “I would be very ill without my metformin-containing medication” (Table 2). Considering the Concerns subscale, the respondents were most worried about the long-term effects of the medicines (51.1%). The Cronbach’s alpha values were 0.82 for the Necessity and 0.86 for the Concerns scales.

**Table 2:** Number and percentage of patients reported Strongly disagree/Disagree/Uncertain and mean (SD) scores for each Necessity and Concerns statement of the BMQ Specific.

Statements	Strongly disagree/Disagree/Uncertain		Total	
	N	%	Mean	SD
**Specific Necessity**			**3.41**	**0.77**
(1) At the moment, my health depends on my metformin-containing medication	254	43.0%	3.56	1.01
(3) My life would be impossible without metformin-containing medication	332	56.2%	3.30	1.04
(5) I would be very ill without my metformin-containing medication	359	60.7%	3.28	0.98
(7) My future health depends on my metformin-containing medication	340	57.7%	3.29	1.05
(10) My metformin-containing medication protects me from feeling worse	245	41.5%	3.60	0.93
**Specific Concerns**			**3.11**	**0.86**
(2) I am worried because I have to take metformin-containing medication	259	43.8%	3.16	1.13
(4) Sometimes I am worried about the long-term effects of my metformin-containing medication	302	51.1%	3.42	1.12
(6) My metformin-containing medication is a mystery to me	242	40.9%	3.19	1.07
(8) My metformin-containing medication makes my life more difficult	162	27.4%	2.76	1.12
(9) Sometimes I am worried that I would become too dependent on my metformin-containing medication	238	40.3%	3.06	1.16
(11) These metformin-containing medicine give me unpleasant side effects	208	35.2%	3.05	1.1

SD: Standard deviation, N: number of respondents

Regarding the Overuse scale almost two-third of the respondents agreed with the answer “doctors prescribe too many medicines”. Among the Harm related questions of the BMQ-General questionnaire, most respondents (46.2%) stated that “people who take medication should stop their treatment for a period of time once in a while”. The Cronbach’s alpha values were 0.74 for the Overuse scale and 0.81 for the Harm scale (see Table 3).

**Table 3:** Number and percentage of patients reported Strongly agree/Agree and mean (SD) scores for each Overuse and Harm statement of the BMQ General.

Statements	Strongly agree/Agree		Total	
	N	%	Mean	SD
**General Overuse**			**3.66**	**0.81**
(1) Doctors prescribe too many medicines	372	62.9%	3.75	1.03
(7) Doctors place too much trust in medicines	344	58.2%	3.59	0.95
(8) If doctors spent more time with patients, they would prescribe fewer medicines.	348	58.9%	3.65	1.04
**General Harm**			**3.18**	**0.80**
(2) People who take medication should stop their treatment for a period of time once in a while	273	46.2%	3.36	1.02
(3) Most of the medicines are addictive	260	44.0%	3.30	1.02
(4) Natural remedies are safer than medicines	224	37.9%	3.25	1.05
(5) Medicines do more harm than good	155	26.2%	2.94	1.04
(6) All medicines are toxic	212	35.9%	3.04	1.15

SD: Standard deviation, N: number of respondents

In the case of diabetes patients, the most common reason for regularly taking or not taking medication was forgetfulness or the intentional reduction of the prescribed medication amount.

As shown in Table 4, mean scores for the exercise (2.55 ± 1.18) and diet (2.55 ± 1.13) scales for the EBAS were higher than scores for the other areas of self-management. Medication barriers had the lowest value (2.16 ± 1.14). The internal reliability was high for all the four scales (Cronbach’s alpha: 0.92-0.93).

**Table 4:** Results on environmental barriers to diabetes-regime adherence scale.

Scales	N	Mean	SD	Cronbach’s alpha
**TOTAL SCORE**	283	2.41	0.73	
**Medication taking**	283	2.16	1.14	0.92
**Exercise**	283	2.55	1.18	0.93
**Glucose testing**	283	2.28	1.13	0.92
**Diet**	283	2.55	1.13	0.93

SD: Standard deviation

### Attitude analysis

**Fig 1:**
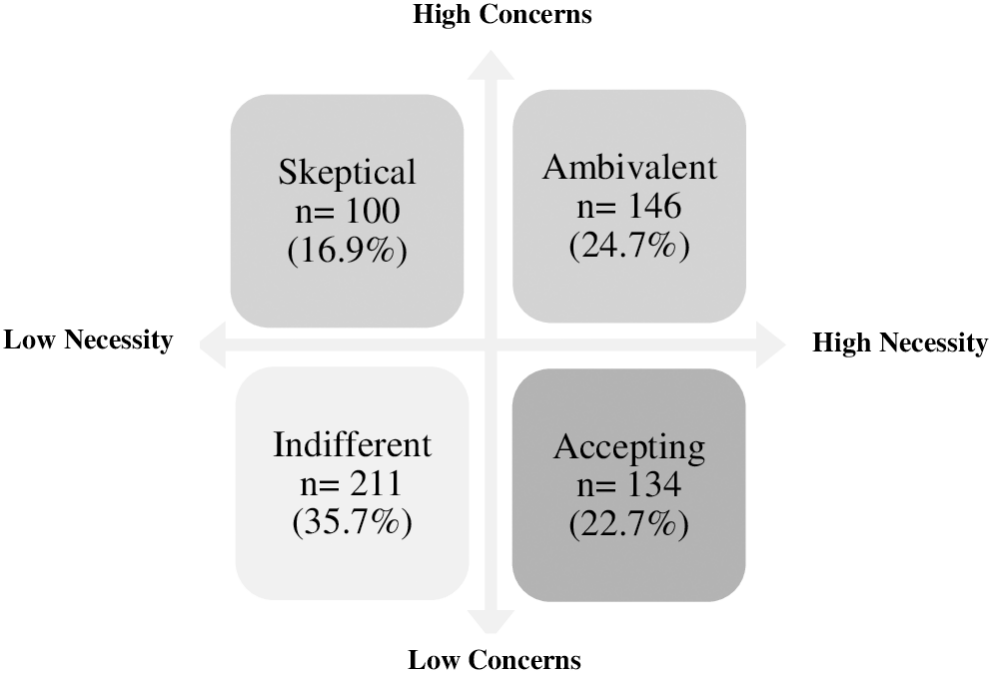
Proportion of respondents in each attitude group with regard to metformin medication in Hungary.

Patients were classified into “attitude groups” based on their beliefs about metformin medication [42]. These groups were created by dividing the median BMQ-Specific Necessity and Concerns scores (Fig 1). The “sceptical” group represented 16.9% of the total respondents. 22.7% of them belonged to the “accepting” group of metformin medication, while the “ambivalent” group comprised 24.7% of participants. The most common type of patient was “indifferent” (35.7%).

### Comparative analysis of the responses

Table 5 shows the results of BMQ multivariate robust regression analysis regarding the Necessity and Concerns scales. Participants who reported a good or very good financial status were more likely to feel the need to take metformin drugs compared to those perceiving bad or very bad financial situation (coef = 0.25; p = 0.020). Those who lived in the Northern Great Plain (coef = -0.29; p = 0.009) region were significantly less concerned about metformin treatment than those who lived in the Pest/Budapest region. Respondents between 55-64 years and those older than 65 were significantly less concerned about metformin medication than those aged 18-24 years (coef = -0.47; p = 0.028 and coef = -0.41; p = 0.047). Participants who reported fair financial status were also less concerned about metformin treatment than those who reported a poor or very poor financial situation (coef = -0.23; p = 0.024).

**Table 5:** Multivariate analysis of the association between socio-demographic variables and Necessity and Concerns scales in the BMQ.

Variables	Necessity				Concerns			
	Coef.	95% CI		p-value	Coef.	95% CI		p-value
**Sex (Ref. Male)**								
Female	-0.01	-0.14	0.13	0.946	-0.03	-0.18	0.12	0.703
**Age group (Ref. 18-24)**								
25-34	0.22	-0.14	0.58	0.230	0.12	-0.28	0.53	0.545
35-44	0.18	-0.18	0.54	0.328	0.12	-0.28	0.53	0.547
45-54	0.10	-0.26	0.47	0.576	-0.27	-0.68	0.14	0.202
55-64	-0.13	-0.50	0.24	0.496	-0.47	-0.89	-0.05	0.028 *
≥65	-0.03	-0.39	0.33	0.861	-0.41	-0.81	-0.01	0.047 *
**Education level (Ref. Primary)**								
Secondary	0.17	-0.18	0.53	0.345	0.19	-0.21	0.58	0.361
Tertiary	0.17	-0.19	0.54	0.349	0.05	-0.36	0.46	0.818
**Marital status (Ref. Other)**								
Married	0.10	-0.05	0.24	0.178	0.00	-0.16	0.16	0.984
**Self-reported financial status (Ref. Bad or very bad)**								
Good or very good	0.25	0.04	0.47	0.020 *	-0.11	-0.35	0.13	0.355
Fair	0.00	-0.17	0.18	0.971	-0.23	-0.43	-0.03	0.024 *
**Healthcare worker (Ref. No)**								
Yes	-0.09	-0.30	0.13	0.417	-0.04	-0.28	0.20	0.723
**Subjective health status (Ref. Fair or poor)**								
Good or better	-0.03	-0.17	0.11	0.661	-0.06	-0.22	0.09	0.437
**Resident population (Ref. 100,000 or more)**								
<1,000	0.14	-0.20	0.49	0.410	-0.38	-0.77	0.01	0.054
1,000-1,999	-0.25	-0.57	0.07	0.119	-0.26	-0.62	0.09	0.149
2,000-4,999	-0.02	-0.24	0.21	0.887	-0.14	-0.40	0.11	0.274
5,000-19,999	-0.12	-0.32	0.09	0.258	-0.19	-0.42	0.04	0.106
20,000-99,999	-0.02	-0.21	0.16	0.812	-0.08	-0.28	0.13	0.464
**NUTS 2 regions (Ref. Pest/Budapest)**								
Central Transdanubia	-0.12	-0.35	0.11	0.289	0.00	-0.26	0.26	0.999
Western Transdanubia	-0.19	-0.46	0.08	0.172	-0.13	-0.43	0.17	0.402
Southern Transdanubia	-0.05	-0.32	0.22	0.714	-0.03	-0.33	0.27	0.839
North Hungary	-0.04	-0.28	0.20	0.748	0.08	-0.19	0.35	0.563
Northern Great Plain	-0.29	-0.51	-0.07	0.009 *	0.13	-0.12	0.37	0.304
Southern Great Plain	0.00	-0.22	0.22	0.993	0.22	-0.02	0.46	0.076

Coef.: Coefficient; CI: Confidence interval; Ref.: Reference groups. The multivariate analysis includes all sociodemographic variables as confounding effects. * Significant finding (p < 0.05)

The results of the BMQ multivariate robust regression analysis for the Harm and Overuse scales are presented in Table 6. Those aged 55-64 years in comparison to those aged 18-24 years were significantly less likely to think that metformin medication was harmful (coef = -0.41; p = 0.037). Participants with secondary education were significantly less likely to think that metformin medication was harmful than those with primary education (coef = -0.50; p = 0.009). Participants who reported a good or very good and fair financial situation were more likely to think that metformin medication was harmful than those who reported a bad or very bad financial situation (coef = -0.23; p = 0.039 and coef = -0.20; p = 0.027). Healthcare workers were more likely to think that taking metformin medication was harmful (coef = -0.25; p = 0.027). Those living in municipalities with a population of 5,000-19,999 were less likely to think that taking metformin medication was harmful than those living in municipalities with a population of more than 100,000 (coef = -0.21; p = 0.045). Those living in the Western Transdanubian region were significantly less likely to think that metformin treatment was harmful than those living in the Pest/Budapest region (coef = -0.34; p = 0.016). Finally, those living in cities with population of 5,000-19,999 were less likely to think that metformin treatment was overused than those living in cities with populations of more than 100,000 (coef = -0.31; p = 0.006).

**Table 6:** Multivariate analysis of the Harm and Overuse scales in the BMQ.

Variables	Necessity				Concerns			
	Coef.	95% CI		p-value	Coef.	95% CI		p-value
**Sex (Ref. Male)**								
Female	-0.11	-0.25	0.03	0.115	0.03	-0.11	0.17	0.673
**Age group (Ref. 18-24)**								
25-34	0.07	-0.30	0.43	0.728	0.04	-0.35	0.43	0.839
35-44	0.03	-0.33	0.40	0.857	-0.01	-0.40	0.38	0.959
45-54	-0.26	-0.63	0.11	0.172	0.14	-0.25	0.53	0.487
55-64	-0.41	-0.79	-0.02	0.037 *	-0.01	-0.41	0.39	0.950
≥65	-0.32	-0.69	0.05	0.087	0.08	-0.31	0.46	0.693
**Education level (Ref. Primary)**								
Secondary	-0.18	-0.54	0.19	0.341	-0.10	-0.48	0.29	0.626
Tertiary	-0.50	-0.87	-0.12	0.009 *	-0.15	-0.54	0.24	0.448
**Marital status (Ref. Other)**								
Married	-0.02	-0.16	0.13	0.844	-0.10	-0.25	0.06	0.215
**Self-reported financial status (Ref. Bad or very bad)**								
Good or very good	-0.23	-0.45	-0.01	0.039 *	-0.11	-0.34	0.12	0.350
Fair	-0.20	-0.38	-0.02	0.027 *	-0.03	-0.22	0.16	0.775
**Healthcare worker (Ref. No)**								
Yes	-0.25	-0.47	-0.03	0.027 *	-0.17	-0.40	0.06	0.151
**Subjective health status (Ref. Fair or poor)**								
Good or better	0.09	-0.06	0.23	0.237	0.07	-0.08	0.22	0.331
**Resident population (Ref. 100,000 or more)**								
<1,000	-0.35	-0.70	0.01	0.054	0.02	-0.35	0.39	0.927
1,000-1,999	0.01	-0.31	0.34	0.931	0.04	-0.31	0.38	0.833
2,000-4,999	-0.03	-0.26	0.20	0.800	0.05	-0.19	0.29	0.689
5,000-19,999	-0.21	-0.42	0.00	0.045 *	-0.31	-0.53	-0.09	0.006 *
20,000-99,999	0.05	-0.14	0.24	0.632	-0.09	-0.29	0.11	0.379
**NUTS 2 regions (Ref. Pest/Budapest)**								
Central Transdanubia	0.15	-0.09	0.38	0.221	0.23	-0.02	0.48	0.070
Western Transdanubia	-0.34	-0.62	-0.06	0.016 *	-0.21	-0.50	0.08	0.148
Southern Transdanubia	-0.07	-0.35	0.20	0.596	0.05	-0.24	0.34	0.732
North Hungary	0.08	-0.17	0.33	0.526	0.09	-0.17	0.35	0.487
Northern Great Plain	-0.02	-0.24	0.20	0.863	0.06	-0.17	0.30	0.592
Southern Great Plain	0.04	-0.18	0.26	0.735	0.13	-0.11	0.36	0.283

Coef.: Coefficient; CI: Confidence interval; Ref.: Reference groups. The multivariate analysis includes all sociodemographic variables as confounding effects. * Significant finding (p < 0.05)

Table 7 shows the results of the multivariate robust regression analysis for the medication and exercise scales of the EBAS. A significant negative association was found for women (coef = -0.32; p < 0.001). In terms of age, those aged 35 or older perceived more barriers to taking medication than those aged 18-24 (35-44: coef = -0.54; p = 0.020; 45-54: coef = -1.15; p < 0.001; 55-64: coef = -1.06; p < 0.001; 65 years or older coef = -1.48; p < 0.001). Those who reported good or better health perceived higher barriers to taking medication than those in fair or poor subjective health status. (coef = -0.20; p = 0.025).

**Table 7:** Multivariate analysis of the association between socio-demographic variables and Medication and Exercise scales in the EBAS.

Variables	Medication				Exercise			
	Coef.	95% CI		p-value	Coef.	95% CI		p-value
**Sex (Ref. Male)**								
Female	-0.32	-0.49	-0.14	<0.001 *	-0.20	-0.40	0.01	0.062
**Age group (Ref. 18-24)**								
25-34	-0.31	-0.77	0.14	0.178	-0.06	-0.60	0.48	0.829
35-44	-0.54	-1.00	-0.09	0.020 *	-0.30	-0.84	0.24	0.269
45-54	-1.15	-1.61	-0.69	<0.001 *	-0.36	-0.90	0.18	0.193
55-64	-1.06	-1.54	-0.58	<0.001 *	-0.38	-0.95	0.18	0.183
≥65	-1.48	-1.95	-1.00	<0.001 *	-1.06	-1.63	-0.50	<0.001 *
**Education level (Ref. Primary)**								
Secondary	-0.46	-1.09	0.18	0.158	-0.78	-1.53	-0.03	0.041 *
Tertiary	-0.39	-1.02	0.25	0.236	-0.74	-1.49	0.02	0.055
**Marital status (Ref. Other)**								
Married	0.05	-0.16	0.25	0.660	0.09	-0.15	0.33	0.471
**Self-reported financial status (Ref. Bad or very bad)**								
Good or very good	-0.15	-0.43	0.13	0.282	-0.48	-0.81	-0.15	0.004 *
Fair	-0.05	-0.30	0.21	0.707	-0.20	-0.50	0.10	0.189
**Healthcare worker (Ref. No)**								
Yes	0.20	-0.06	0.45	0.128	0.17	-0.13	0.47	0.270
**Subjective health status (Ref. Fair or poor)**								
Good or better	-0.20	-0.38	-0.03	0.025 *	-0.34	-0.55	-0.13	0.002 *
**Resident population (Ref. 100,000 or more)**								
<1,000	-0.30	-0.72	0.11	0.153	-0.23	-0.72	0.26	0.351
1,000-1,999	-0.06	-0.55	0.43	0.800	-0.21	-0.79	0.37	0.482
2,000-4,999	-0.14	-0.46	0.17	0.375	0.02	-0.35	0.40	0.909
5,000-19,999	-0.06	-0.33	0.20	0.638	-0.06	-0.38	0.26	0.712
20,000-99,999	-0.14	-0.38	0.10	0.255	0.03	-0.25	0.32	0.829
**NUTS 2 regions (Ref. Pest/Budapest)**								
Central Transdanubia	0.05	-0.25	0.35	0.749	0.04	-0.31	0.40	0.821
Western Transdanubia	-0.25	-0.63	0.13	0.199	0.02	-0.44	0.47	0.940
Southern Transdanubia	-0.126	-0.492	0.24	0.499	-0.152	-0.585	0.281	0.490
North Hungary	-0.223	-0.533	0.087	0.158	-0.117	-0.483	0.25	0.532
Northern Great Plain	0.021	-0.262	0.304	0.885	0.159	-0.176	0.494	0.350
Southern Great Plain	-0.248	-0.521	0.026	0.076	0.045	-0.278	0.369	0.783

Coef.: Coefficient; CI: Confidence interval; Ref.: Reference groups. The multivariate analysis includes all sociodemographic variables as confounding effects. * Significant finding (p < 0.05).

Similarly, we found a significant negative association for several factors (age group, education, self-reported financial status, subjective health status) with exercise among people with diabetes taking metformin. Those aged 65 years or older considered the exercise factor as a greater barrier than those aged 18-24 years (coef = -1.06; p < 0.001). Individuals with secondary education perceived compliance with the exercise factor as a greater obstacle than those with primary education (coef = -0.78; p = 0.041). Those with a good or very good self-reported financial status perceived the need for exercise compared to those with bad or very bad financial status (coef = -0.48; p = 0.004). Among those with diabetes taking metformin in good or better health status we found that they felt more of a barrier to exercise than those in fair or poor health status (coef = -0.34; p = 0.002) (Table 7).

Table 8 shows the results of the multivariate robust regression analysis of the EBAS for the scales regarding glucose test and diet. For the glucose test scale, negative significant association were found for two factors (being a women and older age). Women felt the use of glucose testing as a smaller obstacle than men (coef = -0.22; p = 0.029). Age groups of 45-54, 55-64 and 65 ≤ years or older thought the use of glucose testing as a smaller barrier than the 18-24 years old (45-54: coef = -0.95; p < 0.001; 55-64: coef = -0.88; p = 0.001; 65 ≤ coef = -1.46; p < 0.001).

**Table 8:** Multivariate analysis of the association between socio-demographic variables and Glucose testing and Diet scales in the EBAS.

Variables	Glucose testing				Diet			
	Coef.	95% CI		p-value	Coef.	95% CI		p-value
**Sex (Ref. Male)**								
Female	-0.22	-0.41	-0.02	0.029 *	-0.16	-0.36	0.03	0.093
**Age group (Ref. 18-24)**								
25-34	-0.30	-0.80	0.21	0.249	-0.12	-0.62	0.39	0.648
35-44	-0.47	-0.97	0.04	0.068	-0.34	-0.84	0.16	0.181
45-54	-0.95	-1.46	-0.44	<0.001 *	-0.55	-1.06	-0.05	0.031 *
55-64	-0.88	-1.41	-0.36	0.001 *	-0.42	-0.94	0.11	0.117
≥65	-1.46	-1.98	-0.93	<0.001 *	-1.12	-1.64	-0.60	<0.001 *
**Education level (Ref. Primary)**								
Secondary	-0.33	-1.03	0.37	0.357	-0.18	-0.87	0.52	0.620
Tertiary	-0.14	-0.85	0.56	0.690	-0.03	-0.73	0.67	0.937
**Marital status (Ref. Other)**								
Married	0.05	-0.18	0.27	0.665	-0.03	-0.25	0.20	0.809
**Self-reported financial status (Ref. Bad or very bad)**								
Good or very good	-0.13	-0.44	0.18	0.402	-0.52	-0.83	-0.22	0.001 *
Fair	0.00	-0.28	0.28	0.978	-0.26	-0.53	0.02	0.069
**Healthcare worker (Ref. No)**								
Yes	0.21	-0.07	0.49	0.140	0.29	0.01	0.57	0.044 *
**Subjective health status (Ref. Fair or poor)**								
Good or better	-0.17	-0.37	0.03	0.090	-0.29	-0.48	-0.09	0.004 *
**Resident population (Ref. 100,000 or more)**								
<1,000	-0.23	-0.69	0.23	0.328	-0.42	-0.88	0.03	0.069
1,000-1,999	-0.08	-0.62	0.47	0.778	-0.20	-0.73	0.34	0.471
2,000-4,999	-0.08	-0.43	0.27	0.664	-0.11	-0.46	0.23	0.522
5,000-19,999	-0.15	-0.44	0.15	0.333	-0.20	-0.49	0.09	0.178
20,000-99,999	-0.02	-0.28	0.25	0.898	-0.05	-0.31	0.21	0.709
**NUTS 2 regions (Ref. Pest/Budapest)**								
Central Transdanubia	0.02	-0.31	0.35	0.901	0.22	-0.11	0.55	0.182
Western Transdanubia	0.01	-0.41	0.44	0.951	0.16	-0.27	0.57	0.469
Southern Transdanubia	-0.161	-0.565	0.244	0.435	-0.256	-0.657	0.145	0.210
North Hungary	0.038	-0.305	0.381	0.828	0.058	-0.282	0.398	0.737
Northern Great Plain	0.01	-0.303	0.323	0.949	0.259	-0.051	0.57	0.101
Southern Great Plain	-0.245	-0.548	0.057	0.111	-0.202	-0.502	0.097	0.185

Coef.: Coefficient; CI: Confidence interval; Ref.: Reference groups. The multivariate analysis includes all sociodemographic variables as confounding effects. * Significant finding (p < 0.05)

A significant negative relationship was found for the diet scale for 45-54 year olds (coef = -0.55; p = 0.031) and aged 65 years or older (coef = -1.12; p < 0.001) compared to those aged 18-24. Those reporting good or very good financial status were found to perceive diet adherence as a smaller barrier than those in bad or very bad financial status (coef = -0.52; p = 0.001). Those with good or better subjective health status were less likely to feel that diet adherence was an obstacle than those with fair or poor subjective health status (coef = -0.29; p = 0.004). Only the barriers related to diet showed a significant positive association with being a healthcare worker (coef = 0.29; p = 0.044).

## Discussion

The increase in diabetes prevalence from 2014 to 2021 highlights a growing health concern in Hungary, emphasizing the need for improved diabetes management strategies [1–3]. The higher prevalence of diabetes in older age groups in both studies [26,43] aligns with global trends, where aging populations are more susceptible to type 2 diabetes.

Due to their high internal consistency, the BMQ questionnaire supplemented with the EBAS questionnaire can be used to assess attitudes towards metformin and to identify barriers regarding diabetes self-management. As the influence of beliefs on medication adherence depends on the type of chronic disease [19], it is important to examine these factors using the same measurement tool for all commonly used medications. in the case of the BMQ, all Cronbach values were above 0.7, while in the case of the EBAS, they were above 0.9, thus, all dimensions meet the pre-defined requirements. [41]

In the current study it was found that patients’ age, education level, and perceived financial status impacted their beliefs about medication and their ability to manage diabetes effectively. For instance, older adults (55 years or older) were less concerned about the harmful effects of metformin compared to younger adults (18-24 years). Similarly, patients with secondary education were less likely to view metformin as harmful compared to those with primary education. For the BMQ questionnaire, the main concern of the respondents (51.1%) was related to the long-term effects of their medication. Almost two-thirds agree that doctors prescribe too many drugs. 46.2% believed that drug treatment should be interrupted periodically. Forgetfulness and deliberate reduction of prescribed metformin medication were the most common reasons for patients not adhering to their medication regimen.

In this study adults (35 years or older) saw more barriers to taking medication. Financial status impacts medication necessity, with those in a better financial situation feeling a greater need for metformin. These findings are consistent with other studies that have shown the influence of socio-demographic factors on health behaviours. For example, a study conducted in Eastern India also highlighted the role of demographic factors in medication adherence and diabetes self-management [21]. A Jordanian study [44] supports these findings, emphasizing that socio-economic factors and age are critical in determining medication adherence.

The regression analysis of this study showed that older age and lower education levels were significantly associated with higher barriers to medication and exercise. The study done in Jordan [44] corroborated these findings, demonstrating that socio-demographic factors significantly impact adherence behaviours.

If the patient is taking metformin medication as prescribed, the medicine could effectively treat the disease. The results of our attitude analysis suggest that doctors should pay more attention to patients in the “ambivalent” and “sceptical” groups. Together, the two groups (the “ambivalent” and “sceptical”) account for one third of all patients. These patients are unsure whether they are taking their medicines consistently.

The comparative analysis reveals consistent findings across the sources regarding the impact of environmental barriers and socio-demographic factors on diabetes management. Addressing these barriers through tailored interventions is essential for improving adherence to diabetes regimens and enhancing patient outcomes. Several studies [12,26,43–48] emphasize the need for healthcare providers to consider these factors when developing treatment plans and communicating with patients.

According to research, beliefs about personal models of diabetes and their treatment significantly influence self-management behaviours. It was highlighted that social and environmental factors play a critical role in shaping these beliefs. Participants who perceived higher social support and fewer environmental barriers were more likely to adhere to their treatment regimens [11]. Our study aligns with findings from Hampson et al. [11], demonstrating that environmental barriers and personal beliefs significantly impact diabetes self-management. Both studies emphasize the importance of understanding patient attitudes and the need for tailored interventions to improve adherence.

Environmental barriers significantly affect diabetes self-management, affecting dietary patterns, levels of physical activity, and psychosocial well-being. Overcoming these barriers requires a multifaceted approach that includes policy interventions, community-based initiatives, and health system reform [11]. By promoting a healthy eating habit, improving opportunities for physical activity, and fostering supportive social network, it is possible to create an environment that promotes optimal diabetes self-management and improve the health of individuals affected [32]. Environmental barriers related to diet, exercise, glucose testing, and medications can affect glycaemic control.

In the broader context of diabetes management, research underscores that consistent medication adherence is critical for achieving optimal health outcomes, reducing complications, and minimizing healthcare costs. Diabetes, a chronic condition marked by dysregulated blood glucose levels, is primarily managed through a combination of lifestyle interventions and pharmacotherapy.[12] Medication adherence in diabetes management is multifaceted, involving several behavioural, social, and economic factors. Medication adherence directly influences glycaemic control, which is pivotal in reducing the risk of serious complications like cardiovascular disease, neuropathy, retinopathy, and nephropathy. Non-adherence, whether intentional or unintentional, remains a substantial barrier, often leading to suboptimal health outcomes and higher rates of hospital admissions and emergency visits. Studies consistently highlight a connection between poor adherence and increased morbidity and mortality among diabetes patients, especially those with type 2 diabetes.[32]

Research identifies several key factors that impact adherence to diabetes medication regimens. Patients who understand the importance of their medications and the risks of non-adherence generally have better adherence rates. Complex dosing schedules and polypharmacy (use of multiple medications) are linked to higher rates of non-adherence, as they can be challenging for patients to maintain consistently. Depression, anxiety, and lack of social support can negatively impact adherence. Diabetes itself has a bidirectional relationship with depression, which exacerbates the risk of non-adherence. Cost barriers, including high out-of-pocket costs and inadequate insurance coverage, are significant predictors of medication non-adherence. The quality of patient-provider communication plays a critical role, with empathetic, clear, and supportive interactions leading to higher adherence rates.[36]

Medication adherence in diabetes management is complex, influenced by a range of individual, social, and economic factors. Addressing this issue requires a multifaceted approach that includes patient education, supportive technologies, and policy changes to reduce financial barriers. By promoting adherence, healthcare systems can better manage diabetes at the population level, ultimately improving outcomes and reducing the burden of diabetes-related complications on individuals and healthcare systems alike.

Regular physical activity is essential for managing diabetes effectively. However, environmental barriers such as unsafe neighbourhoods, lack of recreational facilities, and poor urban planning can hinder individuals from engaging in physical activities. Societal attitudes and stigma associated with diabetes can affect an individual’s willingness to seek care and adhere to treatment regimens. Negative perceptions and discrimination can lead to social isolation and psychological distress, which negatively impact diabetes management. The environment, characterized by the affordability and availability of healthy food options, significantly influences eating habits and, consequently, diabetes management. [12]

The current research has some limitations. It was mandatory to answer all the questions for the BMQ and EBAS questionnaires, separately. For some reason, much fewer participants answered all the EBAS questions. A possible explanation could be the length of the questionnaire (EBAS: N = 60; BMQ: N = 19). So, the composition of the representative survey sample was somewhat altered when conducting the statistical analysis (possible explanation could be the length of the questionnaire). Finally, the data collection took place after the COVID-19 pandemic, which may have influenced participants’ attitudes toward medications and environmental barriers regarding diabetes self-management [49].

In conclusion, the internal reliability of the Hungarian BMQ and EBAS was satisfactory based on our examination. There was no BMQ (metformin) and EBAS questionnaire survey in Europe, so this survey can provide a basis for comparison for later European research. Socio-demographic factors have a significant impact on both attitudes to medicine and diabetes self-management. However, the impact varies considerably between different aspects of beliefs and environmental barriers. To further improve medication adherence and self-management among people with diabetes, healthcare professionals should consider relevant socio-demographic factors when communicating with their patients. There is an opportunity to expand on the actionable recommendations for healthcare providers and policy-makers based on the socio-demographic insights presented. The domestic professional guidelines could be updated in such a way that, in doctor-patient communication, we describe who doctors should pay more attention to.

## Supporting information

S1 Data Database. (XLSX)
